# Novel Autoantibodies Related to Cell Death and DNA Repair Pathways in Systemic Lupus Erythematosus

**DOI:** 10.1016/j.gpb.2018.11.004

**Published:** 2019-09-05

**Authors:** Hui Luo, Ling Wang, Ding Bao, Li Wang, Hongjun Zhao, Yun Lian, Mei Yan, Chandra Mohan, Quan-Zhen Li

**Affiliations:** 1 Department of Rheumatology, Xiangya Hospital, Central South University, Changsha 410008, China; 2 Department of Immunology and Internal Medicine, University of Texas Southwestern Medical Center, Dallas, TX 75390, USA; 3 Department of Nephrology, Shanghai Tenth People’s Hospital of Tongji University, Shanghai 200072, China; 4 School of Laboratory Medicine and Life Science, Wenzhou Medical University, Wenzhou 325035, China; 5 Department of Biomedical Engineering, University of Houston, Houston, TX 77004, USA

**Keywords:** Systemic lupus erythematosus, Autoantibodies, ProtoArray, Apoptosis, DNA repair

## Abstract

**Systemic lupus erythematosus** (SLE) is a complex autoimmune syndrome characterized by various co-existing **autoantibodies** (autoAbs) in patients’ blood. However, the full spectrum of autoAbs in SLE has not been comprehensively elucidated. In this study, a commercial platform bearing 9400 antigens (**ProtoArray**) was used to identify autoAbs that were significantly elevated in the sera of SLE patients. By comparing the autoAb profiles of SLE patients with those of healthy controls, we identified 437 IgG and 1213 IgM autoAbs that the expression levels were significantly increased in SLE (*P* < 0.05). Use of the ProtoArray platform uncovered over 300 novel autoAbs targeting a broad range of nuclear, cytoplasmic, and membrane antigens. Molecular interaction network analysis revealed that the antigens targeted by the autoAbs were most significantly enriched in cell death, cell cycle, and **DNA repair** pathways. A group of autoAbs associated with cell **apoptosis** and DNA repair function, including those targeting APEX1, AURKA, POLB, AGO1, HMGB1, IFIT5, MAPKAPK3, PADI4, RGS3, SRP19, UBE2S, and VRK1, were further validated by ELISA and Western blot in a larger cohort. In addition, the levels of autoAbs against APEX1, HMGB1, VRK1, AURKA, PADI4, and SRP19 were positively correlated with the level of anti-dsDNA in SLE patients. Comprehensive autoAb screening has identified novel autoAbs, which may shed light on potential pathogenic pathways leading to lupus.

## Introduction

Autoantibodies (autoAbs) constitute the diagnostic hallmark of autoimmune diseases [1], [2]. AutoAbs play an active role in inflicting target organ pathology through type II and type III hypersensitivity reactions. Hence, defining the autoAbs associated with systemic autoimmune disease has immense significance towards clinical diagnostics and understanding disease pathogenesis [3].

Systemic lupus erythematosus (SLE) is a prototype systemic autoimmune disease which can affect almost every organ in the human body. Appearance of various autoAbs against different antigens is one of the characteristic features of SLE [4]. Thus, serological tests for autoAbs constitute important diagnostic or classification criteria for SLE. However, the etiopathogenesis of this disease remains unclear. Traditional approaches to assay serum autoAbs have typically focused on monitoring the presence of one or a few targeted specificities using defined antigens or tissues as substrates. Thus, immunofluorescence (IF), immunoprecipitation (IP), and enzyme-linked immunosorbent assays (ELISA) remain the dominant platforms used by most clinical and research laboratories that currently assay autoAbs [5]. In recent years, array-based approaches have allowed researchers to multiplex the search for autoAbs in systemic and organ-specific autoimmune diseases. Thus, the application of the autoantigen arrays has greatly facilitated the identification of a wide spectrum of autoAbs that characterize different manifestations of autoimmune diseases [6].

Although autoantigen arrays offer multiplexing, the number of target autoantigens screened has been quite limited (relatively speaking), ranging between 50 and 200 antigens, in most published arrays [7], [8], [9], [10], [11]. More recently, screening for autoAbs using higher density protein arrays has facilitated new autoAb discovery in several diseases [12], [13], [14]. Here, we test the utility of a novel commercial platform bearing 9400 human proteins (ProtoArray), which were expressed in eukaryotic cell lines, to uncover autoAb specificities in SLE. In addition to uncovering ∼400 novel autoAb specificities, the data also shed light on some potential pathogenic pathways that may be important in SLE.

## Results

### Identification of SLE-associated autoAbs — discovery using ProtoArray

**Figure 1:**
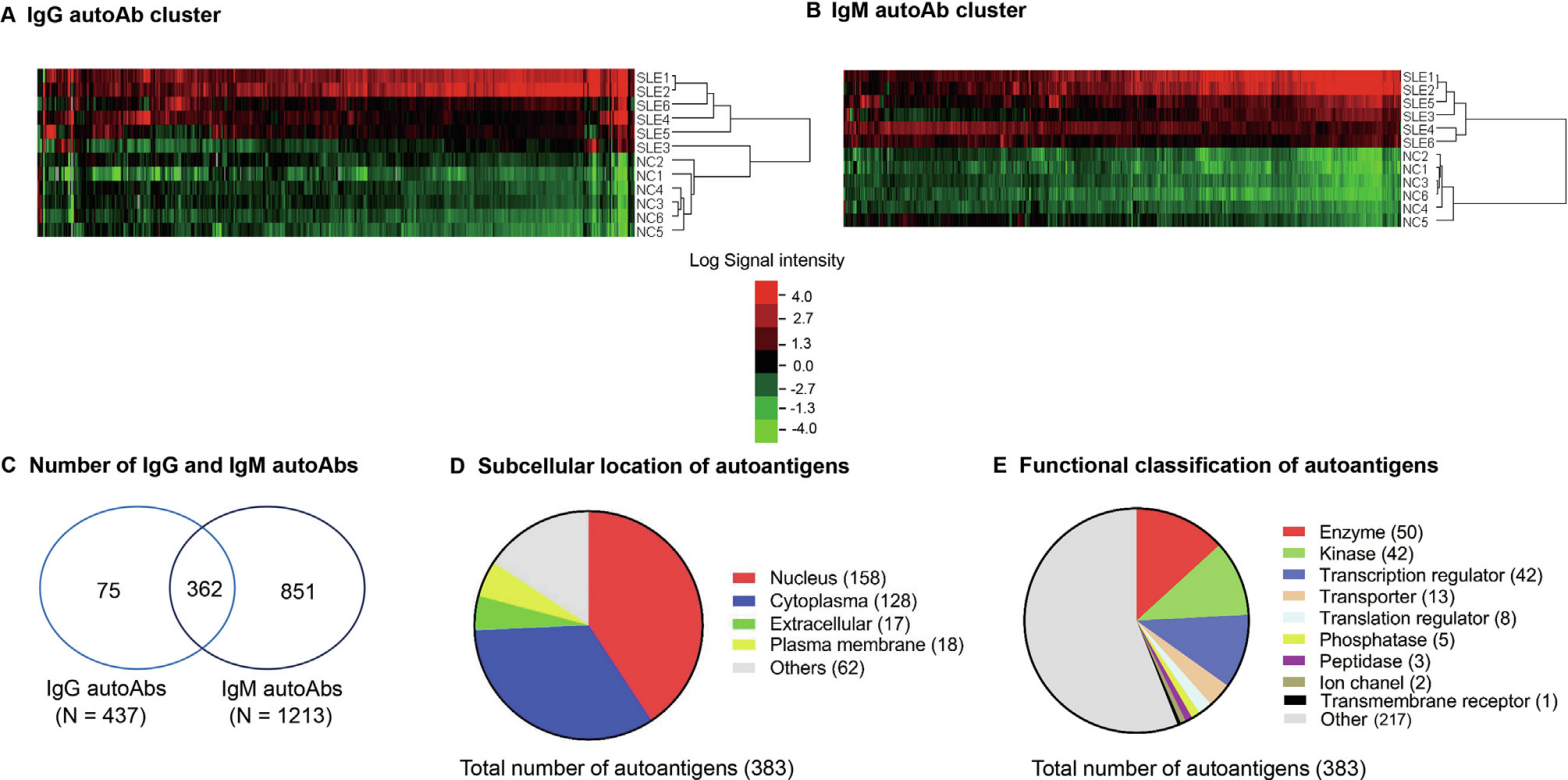
**Clustering and classification of differentially expressed IgG and IgM autoAbs in SLE and NC by ProtoArray** **A.** Clustering and heatmap analyses of the 437 IgG autoAbs with significantly increased levels in SLE (*P* < 0.05; Mann-Whitney U test). Red indicates increased expression and green indicates decreased expression in SLE as compared with NC. **B.** Clustering and heatmap analyses of 1218 IgM autoAbs with significantly-elevated levels in SLE (*P* < 0.05). **C.** Venn diagram of IgG and IgM autoAbs. There are 362 autoAbs commonly detected in both IgG and IgM subtypes. **D.** Subcellular localization of the 383 unique antigens targeted by 437 IgG autoAbs. **E.** Functional classification of the 383 unique autoantigens targeted by IgG autoAbs using IPA. SLE, systemic lupus erythematosus; NC, healthy control; autoAb, autoantibody.

In the discovery phase, we measured the levels of autoAbs targeting 9400 proteins in the sera of SLE (*n* = 12) and healthy controls (NC, *n* = 12) using ProtoArray chips. Every two SLE or NC samples were pooled and hybridized onto one chip and a total of 12 ProtoArray chips were used (6 for SLE and 6 for NC). Statistical analysis revealed a total of 437 IgG autoAbs and 1213 IgM autoAbs, which had significantly elevated levels in SLE patients compared with NC (fold change ≥1.5 and *P* ≤ 0.05) (Figure 1A and B, Table S1, sheets 1 and 2). Among them, 362 autoAbs were common in both IgG and IgM (Figure 1C, Table S1, sheet 3). Although both IgG and IgM autoAbs are associated with SLE, IgG autoAbs are more pathogenic than IgM in the development of SLE [8]. Hence, we focused further analysis on the 437 IgG autoAbs which target 383 unique antigens, including 158 nuclear proteins, 128 cytoplasmic proteins, 18 plasma membrane proteins, 17 extracellular proteins, and 62 unknown specificities (Figure 1D, Table S2). Based on functional annotation using Ingenuity Pathway Analysis (IPA) software, the IgG-targeted proteins included 50 enzymes, 42 kinases, 42 transcription regulators, 13 transporters, 8 translation regulators, 5 phosphatases, 3 peptidases, 2 ion channel proteins, and 1 ligand-dependent nuclear receptor (Figure 1E, Table S2). As expected, about 30 of the IgG autoAbs that were identified to be elevated in SLE using ProtoArray were well-known SLE-specific autoAbs, *e.g.*, anti-DNA, anti-Sjögren’s-syndrome-related antigen A (anti-Ro/SSA), anti-Sjögren syndrome type B antigen (anti-La/SSB), anti-small nuclear ribonucleoproteins (anti-snRNP), anti-histone, and anti-ribosome proteins [6], thus validating the authenticity of the ProtoArray platform for identifying disease-associated autoAbs.

### Validation of SLE-associated autoAbs using customized indirect ELISA

**Figure 2:**
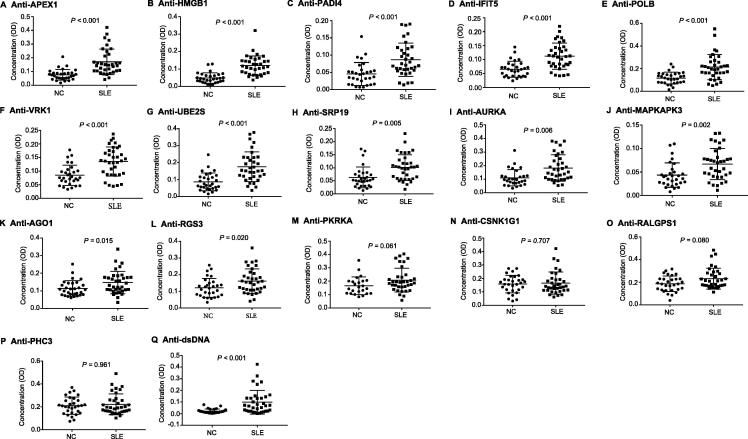
**Validation of the expression of 16 autoAbs using ELISA in a larger cohort of SLE and NC** The levels of 16 most differentially expressed autoAbs involved in apoptosis, cell cycle, and DNA repair pathways were measured using ELISA in a larger cohort of serum samples from SLE patients (*n* = 35) and NC (*n* = 30). Data were analyzed using GraphPad Prism 6.0 software with *P* values of Mann Whitney test presented in each plot. Differences with *P* < 0.05 are considered significant. Of the 16 autoAbs assayed, 12 autoAbs including those targeting APEX1, HMGB1, PADI4, IFIT5, POLB, VRK1, UBE2S, SRP19, AURKA, MAPKAPK3, AGO1, and RGS3, were identified as being increased significantly in SLE than in NC (*P* < 0.05).

Among the significantly upregulated autoAbs identified in SLE using ProtoArray chips, a subset of autoAbs were selected for further validation by ELISA. The validation phase involved a larger cohort of SLE patients (*n* = 35) and NC (*n* = 30). Clinical information pertaining to the subjects included in the discovery and validation phases is listed in Table 1. In total, 16 autoAbs were selected for validation based on three criteria: (1) significantly increased levels in SLE compared with NC (FC > 2, *P* < 0.01); (2) being elevated on both IgG and IgM levels and (3) significantly enriched in the top functional pathways associated with cell death/survival, cell cycle, and DNA recombination, replication, and repair function. Among the 16 autoAbs selected, 9 target nuclear proteins, including APEX nuclease 1, multifunctional DNA repair enzyme (APEX1), ubiquitin-conjugating enzyme E2S (UBE2S), aurora kinase A (AURKA), DNA polymerase beta (POLB), high-mobility group box 1 (HMGB1), vaccinia related kinase 1 (VRK1), regulator of G-protein signaling 3 (RGS3), mitogen-activated protein kinase-activated protein kinase 3 (MAPKAPK3), and polyhomeotic homolog 3 (PHC3), 6 target cytoplasmic proteins, including interferon-inducible double stranded RNA-dependent protein kinase activator A (PRKRA), Ral GEF with PH domain and SH3 binding motif 1 (RALGPS1), peptidyl arginine deiminase, type IV (PADI4), casein kinase 1, gamma 1 (CSNK1G1), argonaute RISC catalytic component 1 (AGO1), and signal recognition particle 19 kDa (SRP19), and 1 targets a plasma membrane protein, Interferon-induced protein with tetratricopeptide repeats 5 (IFIT5) (Table 2). These proteins were classified as kinase, transcription activators, or RNA-binding proteins, based on their functions (Table 2). Further ELISA assays indicated that among the 16 autoAbs tested, the levels of 12 autoAbs targeting APEX1, HMGB1, PADI4, IFIT5, POLB, VRK1, UBE2S, SRP19, AURKA, MAPKAPK3, AGO1 (EIF2C1), and RGS3 remained significantly increased in SLE compared with NC (*P* < 0.05, Figure 2A–L). AutoAb to PRKRA was modestly increased in SLE, with marginal significance (*P* = 0.06, Figure 2M). The remaining three autoAbs (targeting CSNK1G1, RALGPS1, and PHC3) showed no statistically significant difference between SLE and NC (Figure 2N–P). The levels of anti-dsDNA antibody were measured by ELISA as a positive control and showed a significant increase in SLE (Figure 2Q).

**Table 1:** **Profile of study participants**

**Parameter**	**Discovery phase**		**Validation phase**	
	**NC**	**SLE**	**NC**	**SLE**
No. of subjects	12	12	30	35
Age (years)	38.2 ± 5.5	41.7 ± 8.6	35.3 ± 6.1	44.9 ± 7.3
Gender (F/M)	10/2	10/2	25/5	29/6
ANA (EU)	15.2 ± 6.7	47.8 ± 11.3	12.7 ± 4.5	55.6 ± 15.1
ACR criteria	0	6.5 ± 0.4	0	7.4 ± 0.6
Lupus nephritis	–	NA	-	15

*Note*: Age and levels of ANA and ACR were presented as mean ± SD. ACR criteria refer to the scores obtained according to ACR SLE criteria. NC, normal control; SLE, systemic lupus erythematosus; ANA, anti-nuclear antibodies; ACR, American College of Rheumatology; EU, ELISA unit; NA, not available.

**Table 2:** **The 16 upregulated autoAbs in SLE selected for validation using ELISA**

**Protein ID**	**Description**	**Fold change**		**Location**	**Function**
		**IgG**	**IgM**		
APEX1	Apurinic/apyrimidinic endodeoxyribonuclease 1	16.7	181.4	Nucleus	Enzyme
AURKA	Aurora kinase A	12.7	93.1	Nucleus	Kinase
HMGB1	High mobility group box 1	11.5	90.8	Nucleus	GEF
MAPKAPK3	Mitogen-activated protein kinase-activated protein kinase 3	11.8	130.6	Nucleus	Kinase
PHC3	Polyhomeotic homolog 3	195.2	20.5	Nucleus	Transcription regulator
POLB	DNA polymerase beta	11.5	86.7	Nucleus	Enzyme
RGS3	Regulator of G protein signaling 3	7.2	65.6	Nucleus	GTPase activating protein
UBE2S	Ubiquitin conjugating enzyme E2S	16.7	231.3	Nucleus	Enzyme
VRK1	Vaccinia related kinase 1	19.1	234.3	Nucleus	Kinase
AGO1	Argonaute 1, RISC catalytic component	182.7	52.9	Cytoplasm	Translation regulator
CSNK1G1	Casein kinase 1 gamma 1	11.8	49.7	Cytoplasm	Protein kinase
PADI4	Peptidyl arginine deiminase 4	10.2	53.4	Cytoplasm	Enzyme
PRKRA	Protein activator of interferon induced protein kinase EIF2AK2	6.7	45.7	Cytoplasm	Transcription regulator
RALGPS1	Ral GEF with PH domain and SH3 binding motif 1	37.8	17.8	Cytoplasm	Enzyme
SRP19	Signal recognition particle 19	17.7	223.4	Cytoplasm	Enzyme
IFIT5	Interferon induced protein with tetratricopeptide repeats 5	4.8	12.7	Plasma membrane	RNA binding and 7S RNA binding

*Note*: The 16 autoAbs were selected from ProtoArray results for validation based on three criteria: (1) significantly increased levels of autoAbs in SLE (FC > 2, *P* < 0.01); (2) being targeted by both IgG and IgM autoAbs; and (3) enrichment in the top functional pathways associated with cell death/survival, cell cycle, and DNA recombination, replication, and repair function. RISC, RNA-induced silencing complex; EIF2AK2, eukaryotic translation initiation factor 2 alpha kinase 2; GEF, guanine nucleotide exchange factor.

### Detection of serum APEX1 autoAb in SLE patients using Western blotting

**Figure 3:**
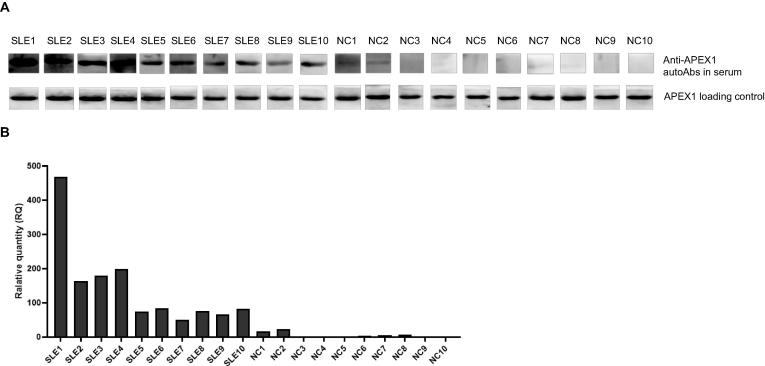
**Detection of anti-APEX1 autoAb in sera of SLE patients using Western blot** **A.** The presence of anti-APEX1 autoAb in SLE patients (*n* = 10) and NC (*n* = 10) detected using Western blot. Recombinant human APEX1 antigen was run on SDS-PAGE and transferred onto PVDF membrane. Sera from SLE or NC (1:500 dilution) were incubated with the strips of membrane for 2 h. After reaction with HRP-conjugated rabbit anti-human IgG, the strips were visualized by chemiluminescent substrate. The upper panel depicts the reaction of autoAb with APEX1 in sera of SLE and NC. The lower panel depicts the binding of the recombinant APEX1 antigen on the membrane with HRP-conjugated rabbit anti-APEX1 antibody as a loading and positive control. **B.** Relative quantity of the band intensities in SLE and NC samples (upper panel in A) and normalized to loading controls (lower panel in A).

To further confirm the expression of the autoAbs identified by ProtoArray, we next measured the presence of anti-APEX1 autoAb by Western blot, in the sera of 10 SLE patients and 10 healthy controls selected based on their anti-APEX1 autoAb titers by ELISA. As expected, all SLE samples with high anti-APEX1 titer by ELISA assays also showed strong binding with APEX1 protein at 35 kD, while weak or no binding was detected in the serum samples from NCs, which were also negative by ELISA (Figure 3).

### Functional classification of autoAbs by pathway analysis

**Figure 4:**
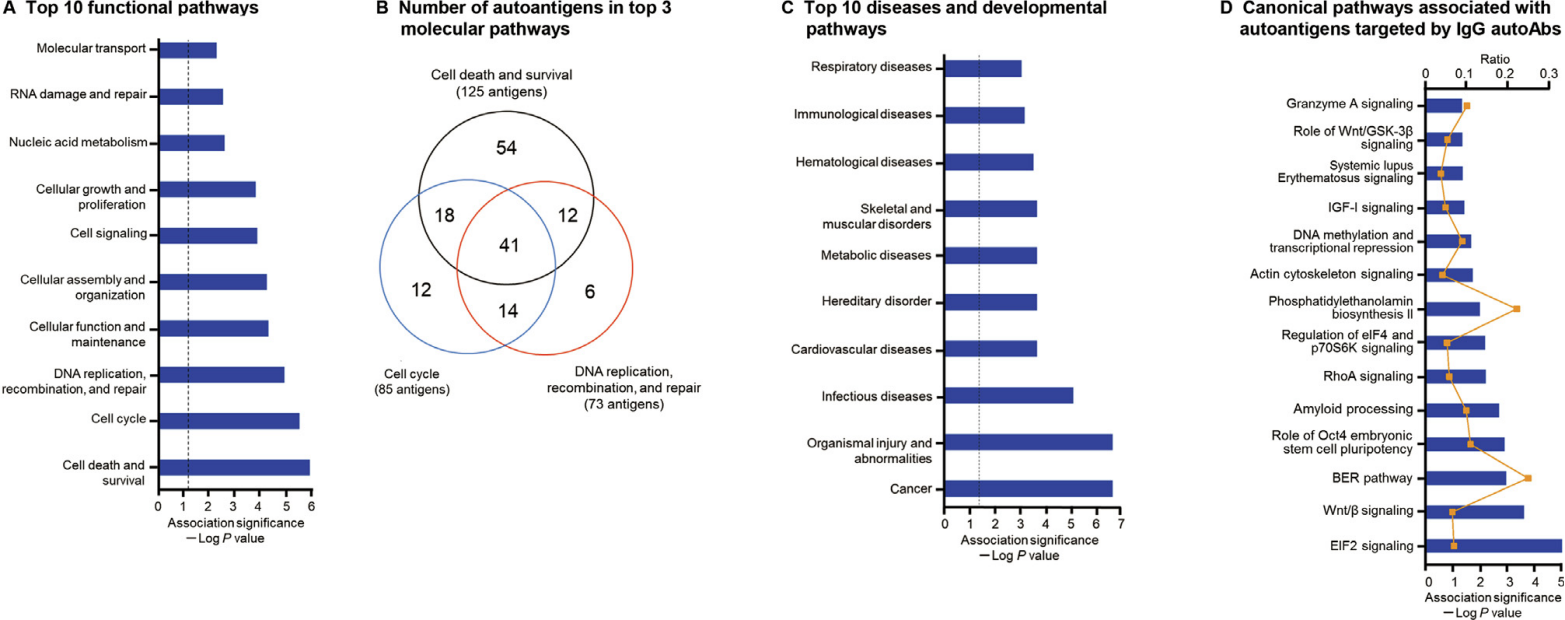
**Pathway and network analyses of differential IgG autoAbs revealed SLE-related pathogenic pathways** **A.** Pathway and network analyses using IPA on the 383 autoantigens targeted by 437 elevated autoAbs revealing the top 10 most significantly enriched functional pathways. Significance of the pathway enrichment was indicated as negative log *P* value (log_10_). The dotted vertical line indicates the significance threshold of *P* = 0.05. **B.** Venn diagram depicting the number of autoantigens involved in the top 3 molecular pathways implicated in cell death and survival, cell cycle, as well as DNA replication, recombination, and repair. **C.** The top 10 diseases and developmental pathways that were significantly enriched in the 383 autoantigens targeted by the 437 IgG autoAbs. **D.** The canonical pathways associated with the 383 autoantigens targeted by the 437 elevated IgG autoAbs. The negative log *P* value and ratio (number of the antoantigens involved in the pathway against the total number of proteins in the pathway) are presented in blue bars and yellow solid lines, respectively.

To explore the functional connections among the autoAbs and their possible association with various autoimmune processes, we performed molecular function and network analysis using IPA software on the 383 autoantigens targeted by the 437 upregulated IgG autoAbs in SLE. A total of 21 molecular interaction networks were identified (Table S3). The top 5 networks that were enriched with the highest numbers of the autoantigens were associated with cell cycle, DNA replication, recombination, and repair, cell death and survival, gene expression, RNA post-transcriptional modification, cancer, cardiovascular disease, cellular assembly and organization, cellular development, as well as cell-to-cell signaling and interactions (Table 3, Figure S1). The most significantly associated functional pathways derived from these networks are shown in Figure 4A and B, including cell death and survival, which involved 125 identified autoantigens (*P* = 6.2 × 10^−7^) (Figure S2A), cell cycle, which involved 85 autoantigens (*P* = 1.6 × 10^−6^) (Figure S2B), and DNA replication, recombination, and repair function, which involved 73 autoantigens (*P* = 6.7 × 10^−6^) (Figure S2C). Several of the autoantigens were related to organismal injury and abnormalities (*P* = 4.39 × 10^−7^), cancer (*P* = 6.7 × 10^−6^), infectious diseases (*P* = 1.3 × 10^−5^), and immunological diseases (*P* = 6.7 × 10^−4^) (Figure 4C). In addition, a number of identified autoantigens have been implicated in some important canonical pathways, including EIF2 signaling (*P* = 8.3 × 10^−6^), Wnt/β-catenin signaling (*P* = 2.2 × 10^−4^) and base extension repair (BER) pathway (*P* = 1.0 × 10^−3^) (Figure 4D).

**Table 3:** **Top diseases and functional networks related to the 383 autoantigens targeted by 437 upregulated IgG autoAbs in SLE**

**ID**	**Proteins in the network**	**Score**	**No. of focus proteins**	**Top diseases and functions**
1	**AMMECR1L, APLF, APOBEC3C, APTX, ARGLU1, ARHGEF5, C1orf174, CCDC28A,** CDK4/6**, CSAG1, EPB41L4A,** ERK1/2, **ERRFI1, GLIPR2, HIRIP3, HIST2H2AC, IPPK, LCE3D, LIG3, MAPKAPK5, MARCH10, MGRN1, MPP5, MTUS1,** Par6**, POLB, RIT1, SEC24C, SLAIN2, SNURF, SRPK2, STX8, TRUB1, VRK2, ZSCAN9**	56	32	Cell cycle, DNA replication, recombination, and repair, as well as gene expression

2	**ASXL1, C8orf33, CDKL3, CLK3, FNDC4, GSPT2, LARP1, LDB2, MAPK8IP2, MRPS6, NCBP3,** NFkB (complex), **NOL7, PELI2, PIH1D3, PPHLN1, RTF1, SAFB2, SARNP, SELENOS, SNRNP70, SOX5, SRP19,** Srp30**, SRPK3, SRSF1, SRSF9, SURF2, TRA2A,** transportin**, UFD1, UTP4, ZC3H14, ZFC3H1, ZNF207**	56	32	RNA post-transcriptional modification, cancer, and cardiovascular disease

3	**ABR,** APC (complex), **AURKA, AURKB, C11orf74, CMSS1, ELOF1, GMNN,** importin beta**, KANSL2, KIF20A, KIF2C, KIFC1, LARP4,** Mapk**, MARK2, MRGBP, MSL3, NEK7, NPM1, NUP50, PADI4,** Plk**, PLK1, PSRC1, RNF126, SRSF2, TCP10L, TPT1, TRMO, TULP3, UBE2S, WDR5, WDR5B, ZC3HC1**	54	31	Cellular assembly and organization, DNA replication, recombination, and repair, as well as cell cycle

4	**AGFG1, BRD3, CACYBP, CSNK2A1, CSNK2A2, DDX54, EDF1,** Eif4g**, FAM90A1, FKBP3, FKBP9, GTF2E2,** holo RNA polymerase II, IκB**, MCTS1, METAP2, NELFE, NMNAT1, OTUD6B,** peptidylprolyl isomerase, phosphatase**, PIP4K2A,** Pkc(s)**, PPAN, PPIL1, PPP1R8, PRKRA, STAU1, SUDS3, TARBP2, TCEA1, VRK1, ZHX1, ZNF574, ZSCAN5A**	48	29	Cell cycle, RNA post-transcriptional modification, and cellular development

5	**ADARB1, AGO1, AGO4,** Akt**, ALOX5, APEX1, BUD23,** c-Src**, CHKA, DDX55,** DNA-PK**,** Eif2**, EIF3G, ERCC6L, FAU, GRB10, HBS1L, HK1, HMGB2, IFRD2, MATK,** MIRLET7**, NOP16, PFKFB3, PIN4, RAB35,** ribosomal 40 s subunit**, RNF4,** Rnr**, RPL35, RPS10, RPS28, RPS27L, SRSF5, TBC1D10C**	46	28	Cell-to-cell signaling and interaction, cancer, as well as cell death and survival

*Note*: Proteins present in the 383 autoantigens are indicated in bold letters. Score for each network was computed by IPA according to the fit of the network to the selected set of focus proteins. The score is derived from a *P* value and indicates the likelihood of the focus proteins in a network being found together due to random chance. Focus proteins refer to the proteins with autoantigens found in the network.

Further IPA analysis was focused on the 16 selected autoantigens which led to the identification of two major molecular interaction networks (Table 4). One of the network with the highest score of association was enriched with 10 autoantigens (APEX1, AURKA, HMGB1, MAPKAPK3, PADI4, POLB, PRKRA, RALGPS1, RGS3, and VRK1) out of a possible 35 molecules associated with cell death and survival, inflammatory response, as well as organismal injury and abnormalities. The second network contained 6 of the identified autoantigens (AGO1, CSNK1G1, IFIT5, PHC3, SRP19, and UBE2S) out of the 35 molecules associated with cancer, organismal injury and abnormalities. In addition, 10 of the 16 autoantigens were associated with cell death/apoptosis/necrosis (Figure S3A), 5 were associated with DNA repair (Figure S3B), and 5 were associated with cell cycle (Figure S3C). Canonical pathway analysis identified the nuclear antigens APEX1 and POLB as critical components of the BER system, which is responsible for maintaining genome integrity by repairing DNA lesions and strand breaks caused by endogenous and exogenous mutagens, such as reactive oxygen species (Figure S3D). Dysfunction of these proteins and the related functional pathways could potentially be associated with the pathogenic processes underlying SLE.

**Table 4:** **Molecular interaction networks related to the 16 upregulated autoAbs in SLE by ProtoArray analysis**

**ID**	**Molecules in Network**	**Score**	**No. of focus molecules**	**Top diseases and functions**
1	AJUBA, ALB, **APEX1**, Arnt-Hif1a, **AURKA**, CRYAB, Efnb dimer, ERK1/2, FKBP5, histone H3, **HMGB1**, KHDRBS1, Mapk, **MAPKAPK3**, MIP1, miR-10, miR-203, miR-21-5p (and other miRNAs with the seed sequence AGCUUAU), NFkB (complex), OGT, OTUD7B, **PADI4**, PASK, **POLB**, POU4F3, **PRKRA**, PRNP, Ral, **RALGPS1**, **RGS3**, SMAD6, SRC, Vegf, **VRK1**	26	10	Cell death and survival, inflammatory response, as well as organismal injury and abnormalities

2	**AGO1**, AGO3, AIP, APP, CBX2, CBX6, CPVL, **CSNK1G1**, DDI1, DDX47, DNA JB11, E2F6, EGFR, **IFIT5**, IPO8, JTB, MDN1, miR-10, miR-30, miR-21-5p (and other miRNAs with the seed sequence AGCUUAU), OTUD7B, PCGF2, PHC1, **PHC3**, PPP5C, PPP6R1, RYBP, SCFD1, **SRP19**, TAX1BP1, TNRC6A, TNRC6B, UBC, **UBE2S**, YAF2	14	6	Cell cycle, cancer, as well as organismal injury and abnormalities

*Note*: Proteins presented in bold letters indicate that they are among the list of the 16 autoAbs which were upregulated both in IgG and IgM in SLE by ProtoArray analysis. Score for each network was computed by IPA according to the fit of the network to the selected set of focus proteins. The score is derived from a *P* value and indicates the likelihood of the focus proteins in a network being found together due to random chance. Focus molecules refer to the molecules targeted by the 16 autoAbs present in the network.

### Correlation of the newly-identified autoAbs with anti-dsDNA autoAb and lupus nephritis

**Figure 5:**
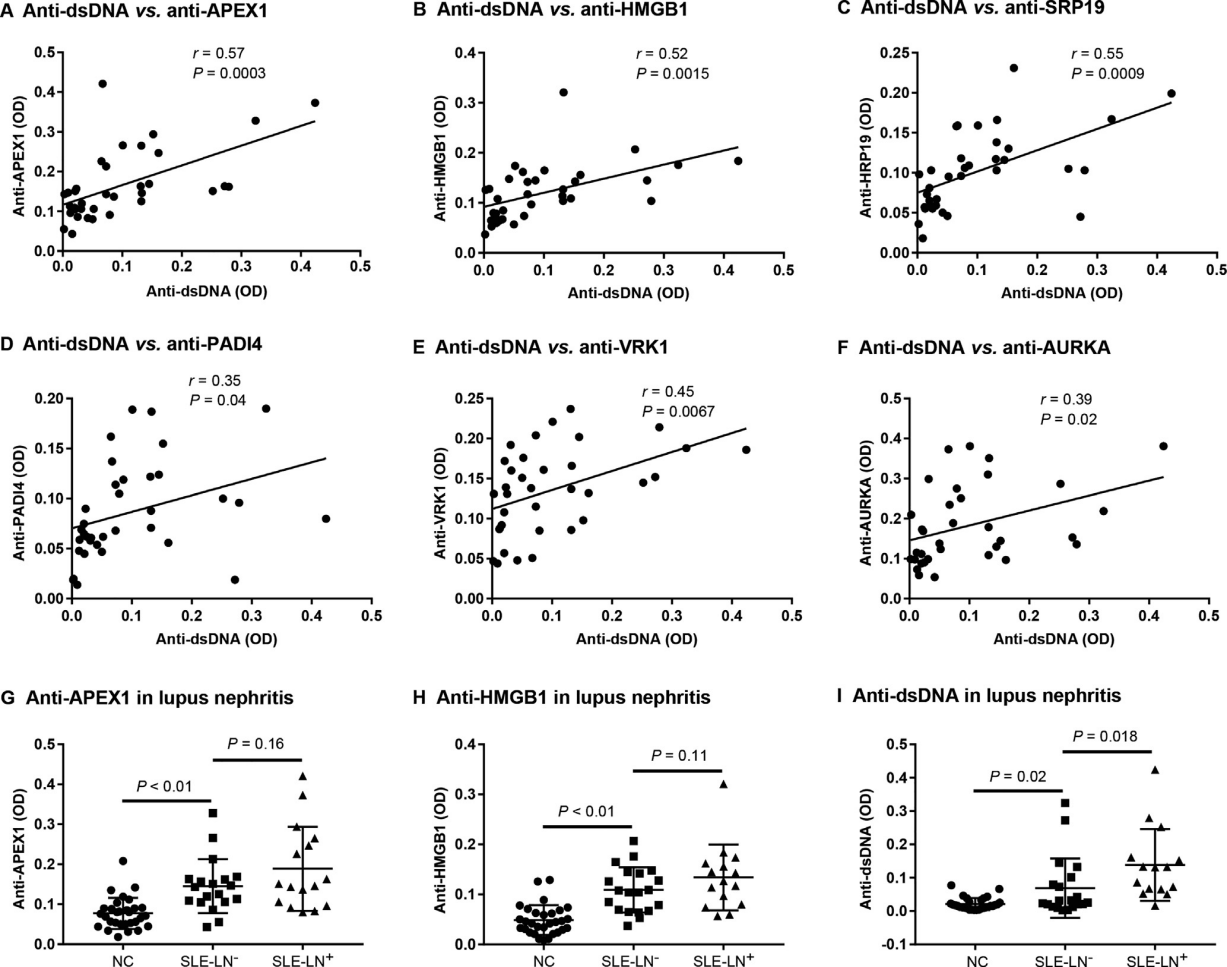
**Correlation of autoAbs with anti-dsDNA and lupus nephritis** The Pearson correlation coefficient of the 16 upregulated autoAbs with anti-dsDNA antibodies was analyzed by GraphPad Prism 6.0 with the levels of autoAbs measured using ELISA from the 35 SLE patients. Pearson's r represents the linear correlation between the levels of the selected autoAb and anti-dsDNA, where 1 means completely positive linear correlation, 0 means no linear correlation, and − 1 means completely negative linear correlation. Correlation with *P* < 0.05 means significant correlation. Six out of the 16 autoAbs were found to be significantly associated with anti-dsDNA, including antibodies to APEX1 (**A**), HMGB1 (**B**), SRP19 (**C**), PADI4 (**D**), VRK1 (**E**), and AURKA (**F**). The levels of anti-APEX1 (G) and anti-HMGB1 (H) autoAbs in NC (*n* = 30), SLE patients with LN (SLE-LN^+^, *n* = 16) and SLE patients without LN (SLE-LN^−^, *n* = 19) were shown in panels **G** and **H**, respectively. Anti-dsDNA was measured as control (**I**). LN, lupus nephritis.

To assess the potential clinical utility of the newly-identified autoAbs, we analyzed the correlation coefficient between the levels of the 16 selected autoAbs and that of anti-dsDNA (which is pathognomonic of SLE) in SLE patients. Among them, levels of six autoAbs showed significant correlation with that of anti-dsDNA antibody. These include anti-APEX1 (*r* = 0.57, *P* = 0.003), anti-HMGB1 (*r* = 0.52, *P* = 0.0015), anti-SRP19 (*r* = 0.55, *P* = 0.0005), anti-PADI4 (*r* = 0.35, *P* = 0.04), anti-VRK1 (*r* = 0.45, *P* = 0.0067), and anti-AURKA (*r* = 0.39, *P* = 0.02) (Figure 5A–F).

We also analyzed the correlation between the levels of the autoAbs and the SLE disease activity index (SLEDAI) score. However, no significant correlation was found between the 16 autoAbs and the SLEDAI score (Table S4).

In order to access if the newly identified auotAbs related with lupus nephritis (LN), one of the most severe complications of SLE, we further compared the levels of the 16 autoAbs between the SLE patients with LN (SLE-LN^+^) and those without LN (SLE-LN^−^) by ELISA. Although no significant difference was identified on the levels of the 16 selected autoAbs between SLE-LN^+^ and SLE-LN^−^, two of the IgG autoAbs (anti-HMGB1 and anti-APEX1) showed a clear trend of increased expression in SLE-LN^+^ compared with SLE-LN^−^ as shown in Figure 5G and H. As a control, the anti-dsDNA antibody level was significantly higher in lupus nephritis patients compared to SLE patients without nephritis (*P* = 0.018, Figure 5I).

## Discussion

The main strength of this study is the simultaneous profiling of thousands of autoAbs against various autoantigens and subsequent their classification into functional groups. SLE is a complex autoimmune syndrome characterized by the co-presence of large numbers of various autoAbs [15]. AutoAbs have been extensively studied as biomarkers of SLE [2], [16]; however, their role in the pathogenesis of SLE and the underlying molecular mechanisms remain poorly understood. In this study, we first performed a global screening of autoAbs in SLE using a commercial planar array (ProtoArray) bearing 9400 functional human proteins. This analysis identified 437 IgG and 1213 IgM autoAbs that were significantly increased in SLE compared to NC. The elevated autoAbs include not only previously well-documented SLE-specific anti-nuclear antibodies, *e.g.*, anti-DNA, anti-Ro/SSA, anti-La/SSB, and anti-Smith ribonucleoproteins (Sm/RNP), but also a large number of novel autoAbs against a broad range of functional proteins, such as DNA repair enzymes, ubiquitin-related enzymes, diverse kinases, transcription regulators, translation initiation factors, interferon-related proteins, and other signaling factors. Further pathway analysis revealed that the antigens targeted by these autoAbs were involved in a wide spectrum of biological functions, including cell cycle, DNA replication, recombination, and repair, cell death and survival, RNA post-transcriptional modification, cancer, cardiovascular disease, cellular assembly and organization, cellular development, and cell-to-cell signaling. Most notably, a large group of upregulated autoAbs in SLE were significantly enriched in the cell death (apoptosis), cell cycle, and DNA repair pathways. Given the fact that impaired clearance of cellular debris is closely associated with the pathogenesis of SLE [17], [18], [19], the autoAbs identified in this study may play potential pathogenic roles by modulating the normal processes of DNA repair, cell death, and clearance of apoptotic debris.

Importantly, the 12 autoAbs which were validated to be increased in SLE all targeted key proteins with important function in cell death and survival, cell cycle, and DNA repair pathways. These include 8 nuclear antigens (HMGB1, APEX1, POLB, VRK1, AURKA, UBE2S, RGS3, and MAPKAPK3) and 4 non-nuclear antigens (AGO1, SRP19, IFIT5, and PADI4). Of these, HMGB1, APEX1, and POLB, are essential components of the BER pathway, which is critical for repairing DNA damage caused by oxidative stress or replication [20], [21], [22]. Dysfunction of this pathway may lead to accumulated cell damage and defective clearance of apoptotic cells, which in turn, may accentuate autoAb production, in a positive feedback loop. Consistent with our result, previous studies have provided evidence supporting the role of HMGB1 and APEX1 in the pathogenesis of SLE. HMGB1 is an important chromatin-binding protein that regulates the binding of transcription factors [23]. Previous reports indicate that anti-HMGB1 may be involved in cell death and inflammatory responses and help to elicit anti-dsDNA antibodies [24], [25], [26], [27]. APEX1, also known as multifunctional DNA repair enzyme 1, is a major apurinic/apyrimidinic endonuclease that is also involved in IL-21-induced signal transduction and B cell activation [28], [29]. As a key enzyme in the BER pathway, APEX1 plays an important role in repairing DNA damage [30]. In addition to HMGB1 and APEX1, POLB is another critical component of the BER pathway [31], but so far, autoAbs against POLB have not been reported. Interestingly, *POLB* gene polymorphisms have been associated with susceptibility to SLE, while decreased POLB activity during the generation of immune diversity was found to elicit lupus-like disease in mice [32], [33]. The identification of these autoAbs targeting key proteins in the BER pathways suggests that autoAbs that potentially interfere with DNA repair may promote anti-nuclear autoimmunity.

Among the novel nuclear autoantigens identified, AURKA, MAPKAPK3, and VRK1 all belong to the serine/threonine protein kinase family, which plays important roles in regulating mitosis, cell cycle progression, and differentiation [34], [35], [36]. AURKA has been found to play a role in experimental autoimmune encephalomyelitis (EAE) by regulating M1 macrophage polarization [34]. Inhibition of AURKA resulted in the production of reactive oxygen species and subsequent triggering of apoptosis. MAPKAPK3 is a key enzyme in the p38 signaling pathway that is activated in response to cellular stress, interleukin-1, and type I interferon [35]. *VRK1* is highly expressed in actively dividing cells and its SNP is associated with SLE skin lesion [36]. Our data show that levels of both AURKA and VRK1 autoAbs were positively correlated with that of anti-dsDNA, implying that the autoAbs against these kinases may play a role in breaching tolerance to DNA in SLE, although the exact molecular mechanisms and sequence of tolerance breaches need to be systematically studied.

AutoAbs against the nuclear proteins UBE2S and RGS3 were also first reported in SLE by this study. UBE2S belongs to the ubiquitin-conjugating enzyme E2 family which functions as an ubiquitin–proteasome for degradation of proteins [37]. Previous studies have shown that UBE2L3, another member of this family, confers risk for SLE by modulating cell proliferation and immune function [38], [39]. Based on the functional property of UBE2S, it is possible that autoAbs to this protein may affect cellular response to DNA damage by interfering with ubiquitin modification at DNA damage sites [37]. In addition, RGS3, a G-protein signaling inhibitor, has been implicated in cell proliferation and apoptosis in cancer [40]. Whether antibodies to this antigen also impact other immune functions or renal disease regulated by G-proteins remains to be elucidated [41], [42].

Other than nuclear antigens, autoAbs against non-nuclear antigens may also play pathogenic roles. In this study, we analyzed autoAbs against 4 non-nuclear antigens: PADI4, IFIT5, SRP19, and AGO1. PADI4 is a member of peptidylarginine deiminase family, which catalyzes the post-translational modification of arginine to citrulline. PADI4 plays a major role in the formation of neutrophil extracellular traps, which is an important source of antigenic nucleic acids in SLE [43]. AutoAbs targeting cyclic citrullinated peptide and PADI4 are prevalent in rheumatoid arthritis [44], [45], [46]. Our study is the first to reveal the presence of anti-PADI4 autoAb in SLE and its correlation with anti-dsDNA, suggesting a pathogenic role for this autoAb in SLE. *IFIT5* belongs to the family of interferon-induced genes that have been defined as signature genes of SLE [47]. As one of the interferon-induced tetratricopeptide repeat family members, IFIT5 enhances IκB kinase (IKK) phosphorylation and NF-κB activation through interacting with transforming growth factor beta-activated kinase 1 (TAK1) and IKK, underscoring the role of IFIT5 in innate immunity [48].

SRP19 is a component of the signal recognition particle (SRP), which is a protein–RNA complex (ribonucleoprotein) functioning in the translocation of protein in the endoplasmic reticulum [49]. AutoAbs against SRPs have been reported as a myositis-specific autoAb (MSA) [50], especially in autoimmune necrotizing myopathy [51], suggesting that this protein may be involved in selected autoimmune processes. Finally, AGO1 was also identified as a potential antigenic target in SLE. AGO1 is a member of Argonaute family which is the primary component of RNA-induced silencing complex (RISC). By direct binding to microRNA, Argonaute proteins play a role in maintaining genome integrity, as well as controlling protein synthesis and RNA stability. Polymorphisms and expression of the *AGO1* and *AGO2* genes have been associated with autoimmune thyroid diseases [52]. Interestingly, autoAbs against AGO2 have been reported in several AIDs, including primary biliary cirrhosis, anti-phospholipid syndrome, and inflammatory myositis [53], [54].

One limitation of this study is that the sample size is relatively small for both the discovery and validation phases. Further validation using large numbers of samples including patients from different ethnic groups is required to delineate the diagnostic utility of the newly-identified autoAbs in this report. Studies are also warranted to ascertain if any of the newly reported IgG autoAbs are associated with specific disease manifestations (in SLE) or evidence of pathogenicity (in animal models). In addition to IgG autoAbs, ProtoArray analysis also revealed a large number of IgM autoAbs. Of note, the co-existence of IgG and IgM autoAbs targeting to the same antigens has been widely reported in SLE [7], [8], [55]. In our earlier studies, the presence of IgM antibodies was associated with milder disease, suggesting that autoAbs of this class may play protective roles [7]. Hence, the significance of specific IgM autoAbs in SLE should be further investigated in future studies.

In conclusion, global autoAb profiling has uncovered a large panel of novel autoAb specificities which may be of diagnostic significance, and may shed light on potential pathogenic pathways in SLE. By functional pathway analysis, autoAbs targeting cell apoptosis and DNA repair pathways are prominent in SLE, and may play a pathogenic role in disease development. Among them, HMGB1, APEX1, AURKA, VRK1, SRP19, and PADI4 and autoAbs to them may play important roles in disease pathogenesis as they represent key proteins (enzymes) in cell cycle and DNA damage repair. Our future study will include cohorts with larger sample size and ethnic diversity for further validation, and also evaluate if the newly-discovered autoAbs and the implicated pathways could be potential therapeutic targets and/or diagnostic markers for SLE.

## Materials and methods

### Serum samples

In total 89 serum samples were obtained from Chinese Han population and from the Department of Rheumatology, Xiangya Hospital, Central South University, China, including 47 samples from SLE patients, 42 from NC. All serum aliquots were stored at −80 °C until used. This study was approved by the Institutional Review Board at Xiangya Hospital, Central South University, China (Changsha, China). Informed consent was obtained in written from all participants included in this study. Clinical information pertaining to these samples is listed in Table 1.

### ProtoArray

ProtoArray Human Protein Microarray V5.0 (Catalog No. PAH052501) bearing over 9400 unique human proteins was purchased from Invitrogen (Carlsbad, CA). All proteins on ProtoArray were recombinant proteins expressed as N-terminal GST-fusion proteins in a eukaryotic cell expression system and printed on an ultrathin layer of nitrocellulose-coated slide (https://www.thermofisher.com). After blocking with protein array blocking buffer (Maine Manufacturing, Sanford, ME) for 30 min, diluted serum samples (1:400) were added onto each array for hybridization at room temperature (RT) for 2 h. After washing with protein array wash buffer (50 mM Tris-Cl, pH 7.5, 150 mM NaCl, and 0.1% Tween 20), Cy3-conjugated anti-human IgG (Catalog No. 109-165-098, Jackson ImmunoResearch, West Grove, PA) and Cy5-conjugated anti-human IgM (Catalog No. 309-585-095, Jackson ImmunoResearch, West Grove, PA) were added as secondary antibodies (1:1000) and incubated at RT for 1 h. The slides were then washed, spin-dried, and scanned using a Genepix 4000B scanner (Molecular Device, San Jose, CA) at 532-nm and 635-nm wavelengths. The images generated were analyzed using Genepix Pro 6.0 software to generate GenePix Report (GPR) files. Statistical analysis was performed using ProtoArray Prospector v5 software to identify autoAbs that were differentially expressed between SLE and NC. Heat maps were generated using Cluster and TreeView software (http://bonsai.hgc.jp/~mdehoon/software/cluster/software.htm).

### ELISA

In total 16 human recombinant proteins, including HMGB1 APEX1, UBE2S, AURKA, POLB, SRP19, IFIT5, RALGPS1 AGO1, RGS3, CSNK1G1, PRKRA, VRK1, PHC3, PADI4, and MAPKAPK3 were purchased from Novus Biologicals (Littleton, CO), R & D Systems (Minneapolis, MN), US Biological (Swampscott, MA), and OriGene Technologies (Rockville, MD), respectively.

Standard indirect ELISA [56], [57] was used to assay the levels of antibodies in human sera against each of the aforementioned proteins. Briefly, proteins (2–4 μg/ml, dissolved in 0.01 M, pH 9.5 sodium bicarbonate buffer) were coated onto Nunc Maxisorp plates precoated with methylated bovine serum albumin (mBSA) (Nunc, Roskilde, Denmark) overnight at 4 °C. After blocking with blocking buffer (10 mM PBS, 3% BSA, 0.1% gelatin, and 3 mM EDTA), human serum (1:100 dilution) was added to each well in duplicate and incubated overnight at 4 °C with agitation. After washing with PBST, alkaline phosphatase-conjugated anti-human IgG (1:5000, Catalog No. 309-055-008, Jackson ImmunoResearch, West Grove, PA) was added to each well and incubated for 2 hr at RT and then p-nitrophenyl phosphate (pNPP) was added as a substrate. OD values were read at 405 nm using an ELx808 microplate reader (BioTek Instruments, Inc. Winooski, VT). The titer of anti-dsDNA autoAb in each serum was also measured by ELISA using the Immulon II plates coated with dsDNA (50 μg/ml) (Dynatech Laboratories, Chantilly, VA) as previously described [58].

### Western blotting

In total 10 μg of APEX nuclease-1 recombinant protein (Catalog No. MBS144352, MyBiosource, San Diego, CA) was run on SDS-PAGE and transferred onto PVDF membrane. Sera from SLE patients and normal controls were diluted 1:500 and added to the membrane and incubated at RT for 2 h. Then HRP-conjugated rabbit anti-human IgG antibody (1:5000, Catalog No. 309-035-008, Jackson ImmunoResearch, West Grove, PA) was added to the membrane for 1 h. Finally, the membranes were incubated with chemiluminescent substrate (Catalog No. CHMM-0060-2C, Surmodics, Eden Prairie, MN) for 1 min and exposed for detection. For visualization of the loading control, the APEX1 transferred PVDF membrane was incubated with HRP-conjugated rabbit anti-human APEX1 antibody (1:2000, Catalog No. LS‑C428306, LSBio, Seattle, WA) for 1 h, followed by chemiluminescent substrate and exposed for detection.

### Statistics and pathway analysis

GraphPad Prism 6.0 software was used for data analysis and graphics. For two-group comparison, an unpaired 2-tailed student’s *t* test was applied. Mann–Whitney test was utilized where data did not fit a normal distribution. Similarly, one-way analysis of variance with Tukey’s multiple comparison tests was used for three or more groups and Kruskal–Wallis test with Dunn’s test was applied to compare all pairs of groups where data did not fit a normal distribution. Differences with *P* < 0.05 were considered significant.

IPA software v7.1 (Ingenuity Systems, Mountain View, CA) was used for network connection and pathway analysis (http://www.ingenuity.com). Proteins were categorized based on molecular function and published interactions with other proteins [59].

## Authors’ contributions

QZL and CM conceived the study. QZL, CM, and HL designed the study. HZ participated in sample collection and data acquisition. MY processed ProtoArray, DB performed Western blotting, and LW (Ling Wang) performed ELISA assays. HL, LW (Li Wang), MY, YL, and QZL performed data analysis. LW (Ling Wang) participated in data interpretation. All authors participated in drafting the manuscript, read, and approved the final version of the manuscript, and gave their consent for publication.

## Competing interests

The authors declare that they have no conflict of interest relating to the conduct of this study or the publication of this manuscript.

## Supplementary material

Supplementary Figure S1 **Top 5 molecular interaction networks derived using the 383 autoantigens targeted by the elevated IgG autoAbs as determined using IPA A.** Network 1 related to cell cycle, DNA replication, recombination and repair, and gene expression. **B.** Network 2 related to RNA post-transcriptional modification, cancer, and cardiovascular disease. **C.** Network 3 related to cellular assembly and organization, cell cycle, DNA replication, as well as recombination and repair. **D.** Network 4 related to cell cycle, RNA post-transcriptional modification, and cellular development. **E.** Network 5 related to cell-to-cell signaling and interaction, cancer, cell death and survival.

Supplementary Figure S2 **The 3 molecular pathways that were enriched in the 383 unique autoantigens targeted by 437 elevated IgG autoAbs in SLE A.** 125 autoantigens targeted by the 437 elevated IgG autoAbs associated with cell death and survival pathways. **B.** 85 autoantigens targeted by the 437 elevated IgG autoAbs associated with cell cycle regulation. **C.** 73 autoantigens targeted by the 437 IgG autoAbs associated with DNA repair, replication, and recombination.

Supplementary Figure S3 **Molecular interaction networks of the 16 IgG autoAbs identified by Protoarray to be increased in both IgG and IgM in SLE by IPA analysis** Ten autoAbs (anti-HMGB1, anti-APEX1, anti-POLB, anti-VRK1, anti-MAPKAPK3, anti-PADI4, anti-RGS3, anti-AGO1 and anti-AURKA) were associated with cell death, necrosis, or apoptosis pathways (**A**), 5 autoAbs (anti-HMGB1, anti-PADI4, anti-PRKRA, anti-POLB and anti-APEX1) were associated with DNA repair function (**B**), and 5 autoAbs (anti-AURKA, anti-PADI4, anti-HMGB1, anti-APEX1 and anti-POLB) were associated with cell cycle progression pathways (**C**). Canonical pathway analysis identified 2 (anti-APEX1 and anti-POLB) out of 16 autoAb associated with the base extension repair pathway. Nodes represent the targeting antigens by respective autoAbs. A direct relation between nodes (proteins) is represented by solid lines and an indirect relation between nodes is represented by dashed lines. Purple circles indicates the protein complex containing the selected autoAb targeting antigens. Values below each autoantigen represent the fold change of the autoAb in SLE *vs*. NC.
