# Supplementary material for: Novel Autoantibodies Related to Cell Death and DNA Repair Pathways in Systemic Lupus Erythematosus
Source: Genomics Proteomics Bioinformatics. 2019 Sep 5;17(3):248–59. doi: 10.1016/j.gpb.2018.11.004 (PMC6818352; doi:10.1016/j.gpb.2018.11.004)
Supplement: Supplementary Figure S1 — Top 5 molecular interaction networks derived using the 383 autoantigens targeted by the elevated IgG autoAbs as determined using IPA A. Network 1 related to cell cycle, DNA replication, recombination and repair, and gene expression. B. Network 2 related to RNA post-transcriptional modification, cancer, and cardiovascular disease. C. Network 3 related to cellular assembly and organization, cell cycle, DNA replication, as well as recombination and repair. D. Network 4 related to cell cycle, RNA post-transcriptional modification, and cellular development. E. Network 5 related to cell-to-cell signaling and interaction, cancer, cell death and survival. [file mmc1.pdf]

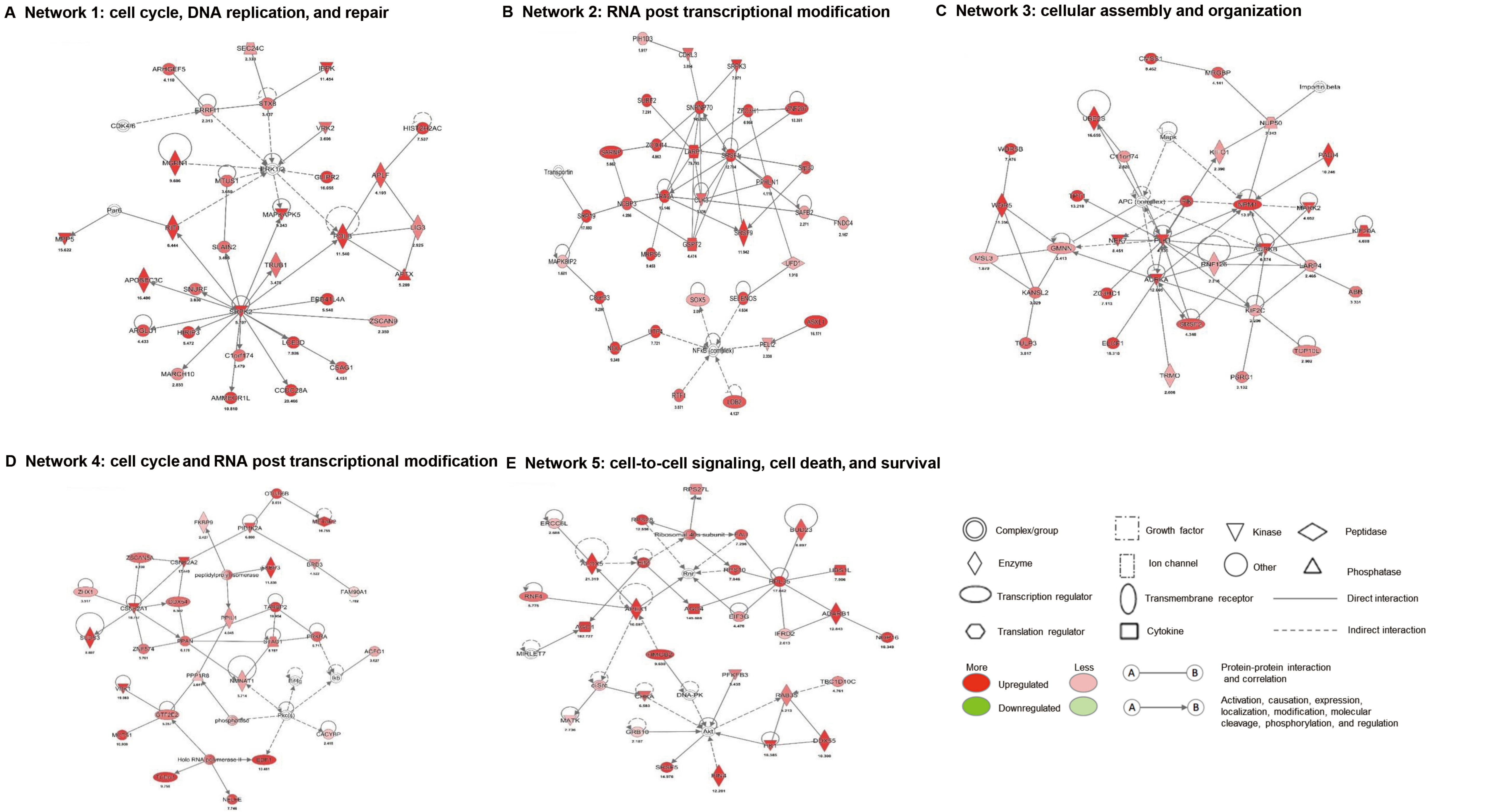
A Network 1: cell cycle, DNA replication, and repair
B Network 2: RNA post transcriptional modification
C Network 3: cellular assembly and organization
D Network 4: cell cycle and RNA post transcriptional modification
E Network 5: cell-to-cell signaling, cell death, and survival
Complex/group
Enzyme
Transcription regulator
Translation regulator
Growth factor
Ion channel
Transmembrane receptor
Cytokine
Kinase
Other
Peptidase
Phosphatase
Direct interaction
Indirect interaction
More
Less
Upregulated
Downregulated
Protein-protein interaction and correlation
Activation, causation, expression, localization, modification, molecular cleavage, phosphorylation, and regulation
