# Supplementary material for: Novel Autoantibodies Related to Cell Death and DNA Repair Pathways in Systemic Lupus Erythematosus
Source: Genomics Proteomics Bioinformatics. 2019 Sep 5;17(3):248–59. doi: 10.1016/j.gpb.2018.11.004 (PMC6818352; doi:10.1016/j.gpb.2018.11.004)
Supplement: Supplementary Figure S2 — The 3 molecular pathways that were enriched in the 383 unique autoantigens targeted by 437 elevated IgG autoAbs in SLE A. 125 autoantigens targeted by the 437 elevated IgG autoAbs associated with cell death and survival pathways. B. 85 autoantigens targeted by the 437 elevated IgG autoAbs associated with cell cycle regulation. C. 73 autoantigens targeted by the 437 IgG autoAbs associated with DNA repair, replication, and recombination. [file mmc2.pdf]

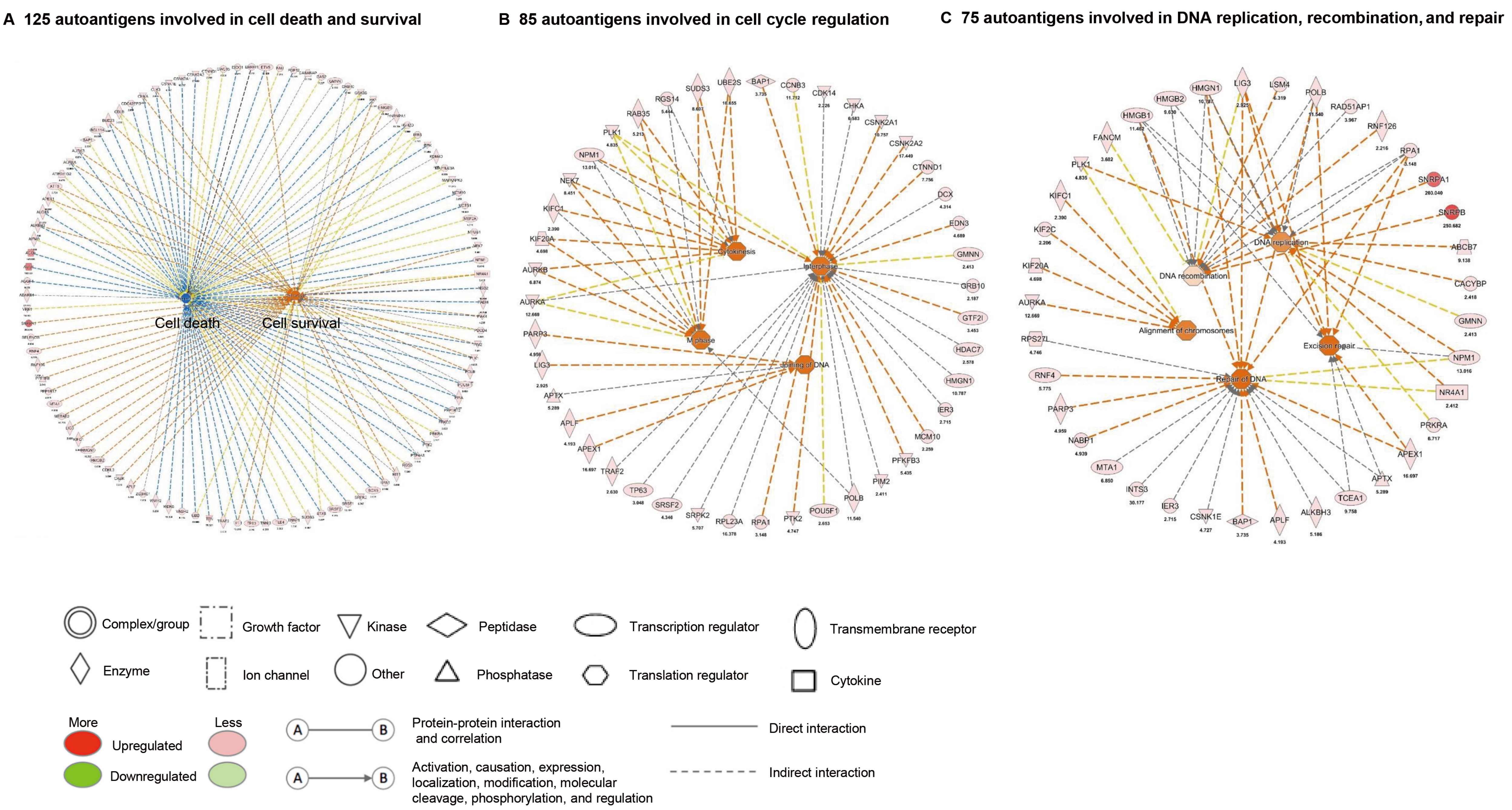
A 125 autoantigens involved in cell death and survival
Cell death
Cell survival
B 85 autoantigens involved in cell cycle regulation
Cytokinesis
Interphase
M phase
Joining of DNA
SUDS3 8.607
UBE2S 16.655
BAP1 3.735
CCNB3 11.712
CDK14 2.226
RGS14 5.444
RAB35 5.213
PLK1 4.835
NPM1 13.016
NEK7 8.451
KIFC1 2.390
KIF20A 4.698
AURKB 6.874
AURKA 12.669
PARP3 4.959
LIG3 2.925
APTX 5.289
APLF 4.193
APEX1 16.697
TRAF2 2.630
TP63 3.048
SRSF2 4.346
SRPK2 5.707
RPL23A 16.378
RPA1 3.148
PTK2 4.747
POU5F1 2.653
POLB 11.540
PIM2 2.411
PFKFB3 5.435
MCM10 2.259
IER3 2.715
HMGN1 10.787
HDAC7 2.578
GTF2I 3.453
GRB10 2.187
GMNN 2.413
EDN3 4.689
DCX 4.314
CTNND1 7.756
CSNK2A2 17.449
CSNK2A1 10.757
CHKA 6.583
C 75 autoantigens involved in DNA replication, recombination, and repair
DNA replication
DNA recombination
Alignment of chromosomes
Excision repair
Repair of DNA
HMGN1 10.787
LIG3 2.925
LSM4 6.319
POLB 11.540
RAD51AP1 3.967
RNF126 2.216
RPA1 3.148
SNRPA1 203.040
SNRPB 250.682
ABCB7 9.138
CACYBP 2.418
GMNN 2.413
NPM1 13.016
NR4A1 2.412
PRKRA 6.717
APEX1 16.697
APTX 5.289
TCEA1 9.758
ALKBH3 5.186
APLF 4.193
BAP1 3.735
CSNK1E 4.727
IER3 2.715
INTS3 30.177
MTA1 6.850
NABP1 4.939
PARP3 4.959
RNF4 5.775
RPS27L 4.746
AURKA 12.669
KIF20A 4.698
KIF2C 2.206
KIFC1 2.390
PLK1 4.835
FANCM 3.682
HMGB1 11.462
HMGB2 9.630
Complex/group
Growth factor
Kinase
Peptidase
Transcription regulator
Transmembrane receptor
Enzyme
Ion channel
Other
Phosphatase
Translation regulator
Cytokine
More
Less
Upregulated
Downregulated
Protein-protein interaction and correlation
Activation, causation, expression, localization, modification, molecular cleavage, phosphorylation, and regulation
Direct interaction
Indirect interaction
