# Supplementary material for: Novel Autoantibodies Related to Cell Death and DNA Repair Pathways in Systemic Lupus Erythematosus
Source: Genomics Proteomics Bioinformatics. 2019 Sep 5;17(3):248–59. doi: 10.1016/j.gpb.2018.11.004 (PMC6818352; doi:10.1016/j.gpb.2018.11.004)
Supplement: Supplementary Figure S3 — Molecular interaction networks of the 16 IgG autoAbs identified by Protoarray to be increased in both IgG and IgM in SLE by IPA analysis Ten autoAbs (anti-HMGB1, anti-APEX1, anti-POLB, anti-VRK1, anti-MAPKAPK3, anti-PADI4, anti-RGS3, anti-AGO1 and anti-AURKA) were associated with cell death, necrosis, or apoptosis pathways (A), 5 autoAbs (anti-HMGB1, anti-PADI4, anti-PRKRA, anti-POLB and anti-APEX1) were associated with DNA repair function (B), and 5 autoAbs (anti-AURKA, anti-PADI4, anti-HMGB1, anti-APEX1 and anti-POLB) were associated with cell cycle progression pathways (C). Canonical pathway analysis identified 2 (anti-APEX1 and anti-POLB) out of 16 autoAb associated with the base extension repair pathway. Nodes represent the targeting antigens by respective autoAbs. A direct relation between nodes (proteins) is represented by solid lines and an indirect relation between nodes is represented by dashed lines. Purple circles indicates the protein complex containing the selected autoAb targeting antigens. Values below each autoantigen represent the fold change of the autoAb in SLE vs. NC. [file mmc3.pdf]

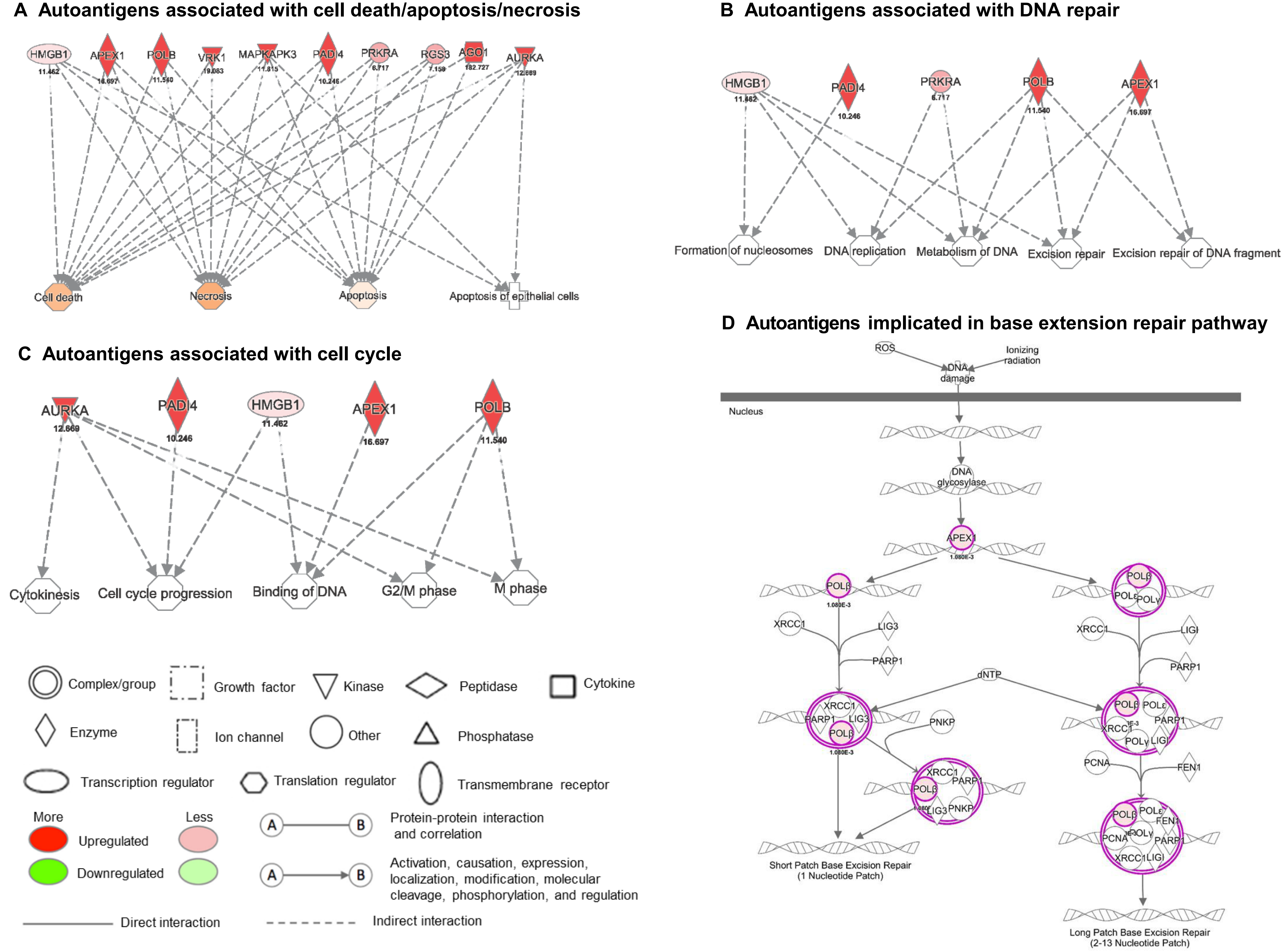
A Autoantigens associated with cell death/apoptosis/necrosis
HMGB1
11.462
APEX1
16.697
POLB
11.540
VRK1
19.083
MAPKAPK3
11.815
PADI4
10.246
PRKRA
6.717
RGS3
7.158
AGO1
182.727
AURKA
12.669
Cell death
Necrosis
Apoptosis
Apoptosis of epithelial cells
B Autoantigens associated with DNA repair
HMGB1
11.462
PADI4
10.246
PRKRA
6.717
POLB
11.540
APEX1
16.697
Formation of nucleosomes
DNA replication
Metabolism of DNA
Excision repair
Excision repair of DNA fragment
C Autoantigens associated with cell cycle
AURKA
12.669
PADI4
10.246
HMGB1
11.462
APEX1
16.697
POLB
11.540
Cytokinesis
Cell cycle progression
Binding of DNA
G2/M phase
M phase
D Autoantigens implicated in base extension repair pathway
ROS
Ionizing radiation
DNA damage
Nucleus
DNA glycosylase
APEX1
1.080E-3
POLβ
1.080E-3
POLβ
POLε
POLγ
XRCC1
LIG3
PARP1
XRCC1
LIGI
PARP1
dNTP
XRCC1
PARP1
LIG3
POLβ
1.080E-3
PNKP
POLβ
POLε
XRCC1
PARP1
POLγ
LIGI
XRCC1
PARP1
POLβ
LIG3
PNKP
PCNA
FEN1
POLβ
POLε
FEN1
PCNA
POLγ
PARP1
XRCC1
LIGI
Short Patch Base Excision Repair
(1 Nucleotide Patch)
Long Patch Base Excision Repair
(2-13 Nucleotide Patch)
Complex/group
Growth factor
Kinase
Peptidase
Cytokine
Enzyme
Ion channel
Other
Phosphatase
Transcription regulator
Translation regulator
Transmembrane receptor
More
Less
Upregulated
Downregulated
Protein-protein interaction and correlation
Activation, causation, expression, localization, modification, molecular cleavage, phosphorylation, and regulation
Direct interaction
Indirect interaction
